# French and English Opinions on the Treatment of Typhus

**Published:** 1845-09

**Authors:** 


*French and English opinions on the Treatment of Typhus.*—In the treatment of our fevers, we have daily occasion to witness the pernicious effects of an irrational practice. There are still many physicians amongst us who almost invariably have recourse to the use of bloodletting and other debilitant remedies. The effect of such a course is inevitably to render the convalescence exceedingly tedious and often imperfect. Many simple cases are thus converted into very troublesome and unmanageable ones, in consequence of the impaired energy of the vital forces that has been induced. When the occurrence of the symptoms of adynamic weakness forces such practitioners to discontinue the use of their lowering regimen, they generally resort to the use of blisters and the administration of tonic medicines.

Let us briefly review some of the features of the disease.

There is usually a more or less considerable derangement of the stomach and bowels at first. Fortunately we have a remedy, or rather a class of remedies, which is exactly suited for the relief of such symptoms—we allude to emeto-cathartics: they generally act almost magically in removing the symptoms of the first stage of such fevers. After due evacuation both upwards and downwards, not only does the pyrexia usually subside, but all the phenomena of congestion and local irritation, that may have been present, are more or less completely relieved. The same decided and prompt benefit cannot indeed be expected from the use of these remedies, if the fever has existed two or three days before they have been exhibited, and if any delirium be present: but even then the relief is sometimes very notable; the headache, stupor, and general prostration not unfrequently ceasing, or, at all events, being very materially diminished, after the action of the vomiting has entirely ceased.—*Gazette Medicale, Aout,* 1844.

We cordially assent to the practical truth and importance of these therapeutic instructions respecting the use of Emetics and Purgatives in the first stage of most typhoid fevers. We have often expressed our own opinions on this subject in the pages of this Journal, and strenuously urged our readers to recur to the practice recommended by the older physicians, and most religiously to eschew the evil ways of such advisers as the disciples of the Broussain school. Still, we are not inclined to go quite so far as our French brother, when he goes on to advise the administration (however guarded) of emeto-cathartic medicines in almost all stages of the fever of which he is treating. The paragraph, to which we object, runs thus:—"At a more advanced period of the disease, when the patients have fallen into an adynamic state, we must be more reserved in the exhibition of these remedies; not that they are not absolutely required or allowable; but only because the state of extreme debility usually present demands that the patient's strength be somewhat revived before they be given. Such cases require the use of stimulants—such as blisters, sinapisms to the abdomen and extremities—before we have recourse to the *heroic remedy*. However, as soon as the patient's strength is recovered, we should not delay longer, but resort at once to the exhibition of an emeto-cathartic: it is the sure means of preventing a fatal tendency." This seems to us dangerous advice. It is very rarely judicious to administer emetics in the advanced stage of febrile affections, when there is considerable prostration of strength; unless, indeed, the symptoms of gastric derangement are very obvious, and Nature herself makes an effort to get rid of the peccant secretions of the stomach and duodenum by the way of vomiting. The present is one out of many instances that might be adduced to show how liable the French practitioners are to carry everything to extremes. A few years ago, the mere mention of an emetic in Typhus fever—which at that time was of necessity a *gastro-enterite*—would have been denounced by nine-tenths of the physicians in Paris as incendiary and most dangerous practice. Now-a-days there seems to be a tendency to run to the other end of the race-course; and we should not be at all surprised to hear of ipecacuanha and tartar-emetic taking the heroic seat which has been so long occupied by leeches and "boissons adoucissantes."—*Med. Chur. Review.*

